# Supplementary material for: Deciphering regulatory DNA sequences and noncoding genetic variants using neural network models of massively parallel reporter assays
Source: PLoS One. 2019 Jun 17;14(6):e0218073. doi: 10.1371/journal.pone.0218073 (PMC6576758; doi:10.1371/journal.pone.0218073)
Supplement: S1 Fig — (A) Correlation for fragments tested in K562 cells using the minimal promoter (minP); same plot as Fig 1C with added marginal distributions. (B) K562 cells with the SV40P promoter. (C) HepG2 cells with minP. (D) HepG2 cells with SV40P. (PDF) [file pone.0218073.s001.pdf]

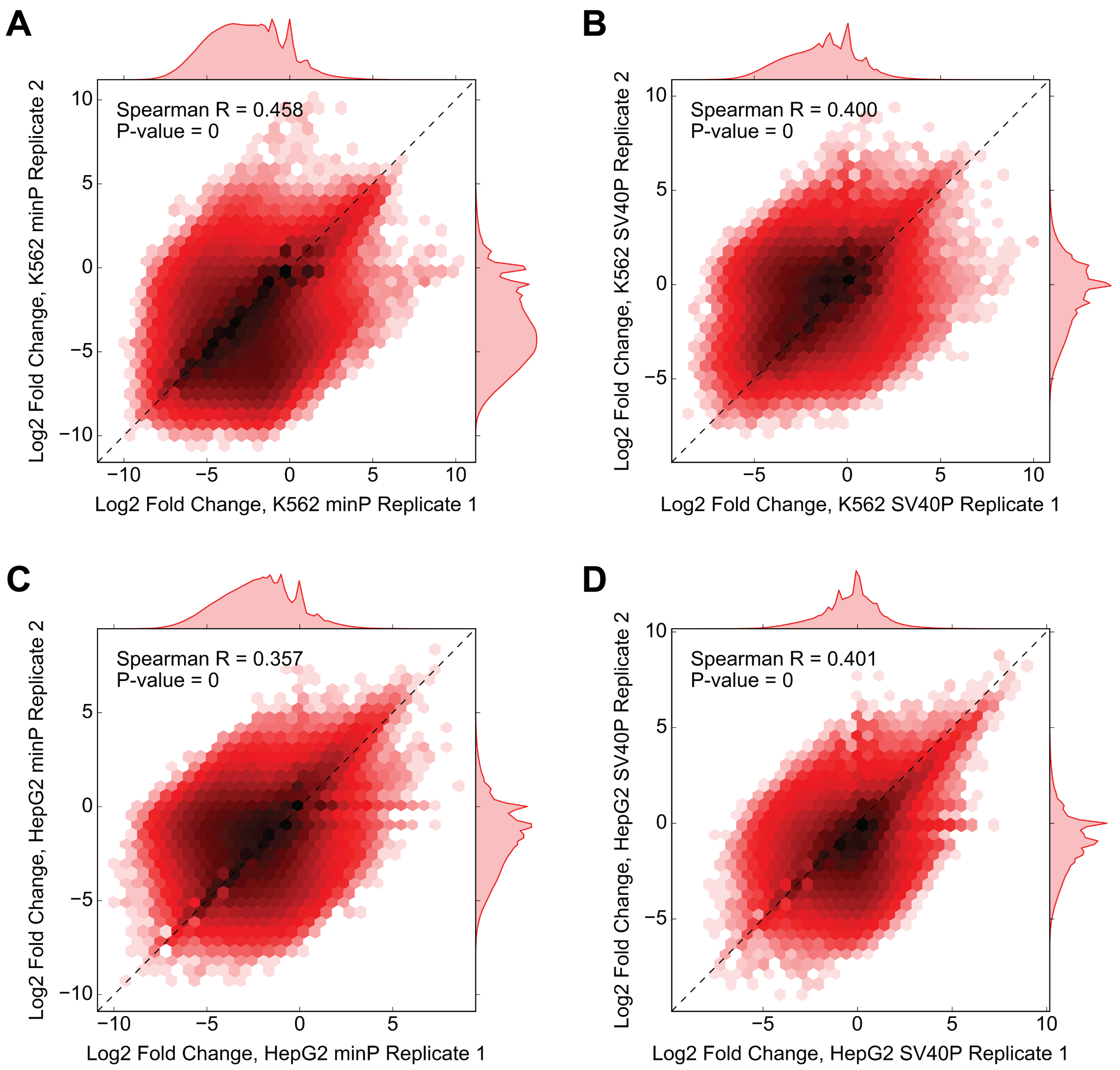

Supplementary Figure 1: Between-replicate correlations for the four experimental groups in Sharpr-MPRA.
(A) Correlation for fragments tested in K562 cells using the minimal promoter (minP); same plot as **Figure 1C** with added marginal distributions. *P*-values listed as 0 are less than Python's float precision, i.e. $P < 1E\text{-}300$.
(B) K562 cells with the SV40P promoter.
(C) HepG2 cells with minP.
(D) HepG2 cells with SV40P.
