# Supplementary material for: Deciphering regulatory DNA sequences and noncoding genetic variants using neural network models of massively parallel reporter assays
Source: PLoS One. 2019 Jun 17;14(6):e0218073. doi: 10.1371/journal.pone.0218073 (PMC6576758; doi:10.1371/journal.pone.0218073)
Supplement: S2 Fig — (A) Correlation between experimental regulatory activity z-scores and predicted regulatory activity z-scores for the K562 SV40P task (analogous to Fig 2A). These predictions are for fragments in the held-out test set (Sharpr fragments in chromosome 18). (B) Performance for HepG2 minP task. (C) Performance for HepG2 SV40P task. (D) Positive correlation between the difference in regulatory activity across replicates vs. prediction error, i.e., fragments with more noisy experimental values have reduced prediction accuracy. (E) Improved prediction performance (ρ = 0.57) for fragments lying in accessible putative enhancers designated as ‘DnaseD’ or ‘FaireW’ states by ChromHMM annotations for the K562 cell type. (PDF) [file pone.0218073.s002.pdf]

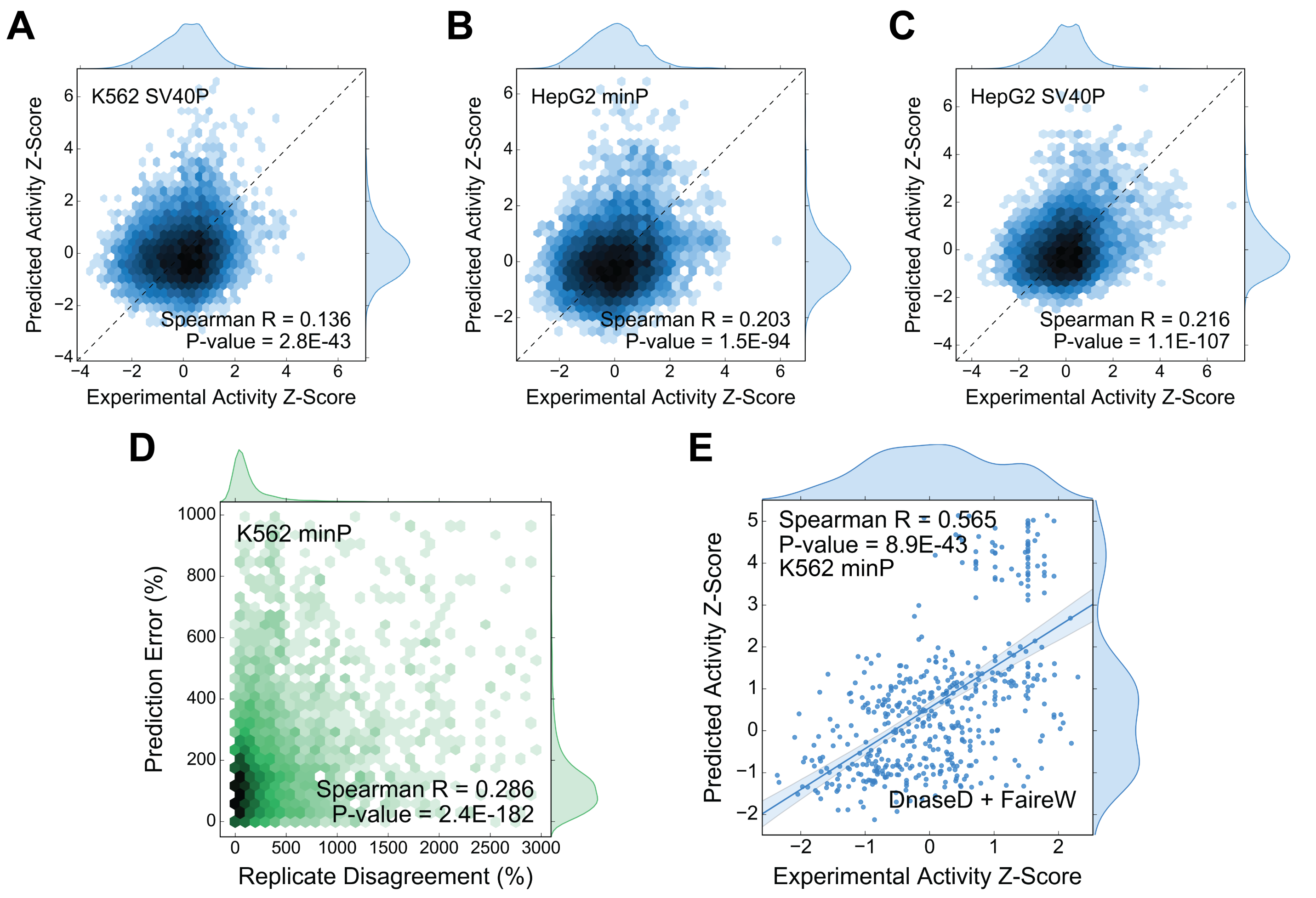

Supplementary Figure 2: Detailed look at MPRA-DragoNN's prediction performance.
(A) Correlation between experimental regulatory activity z-scores and predicted regulatory activity z-scores for the K562 SV40P task (analogous to **Figure 2A**). These predictions are for fragments in the held-out test set (Sharpr fragments in chromosome 18).
(B) Performance for HepG2 minP task.
(C) Performance for HepG2 SV40P task.
(D) Positive correlation between the difference in regulatory activity across replicates vs. prediction error, *i.e.*, fragments with more noisy experimental values have reduced prediction accuracy.
(E) Improved prediction performance (ρ = 0.57) for fragments lying in accessible putative enhancers designated as 'DnaseD' or 'FaireW' states by ChromHMM annotations for the K562 cell type.
