# Supplementary material for: Deciphering regulatory DNA sequences and noncoding genetic variants using neural network models of massively parallel reporter assays
Source: PLoS One. 2019 Jun 17;14(6):e0218073. doi: 10.1371/journal.pone.0218073 (PMC6576758; doi:10.1371/journal.pone.0218073)
Supplement: S3 Fig — (A) Correlation between averaged DeepLIFT vs. averaged SHARPR nucleotide scores at each of the 328K motif matches that overlap at least one Sharpr-MPRA fragment. Each datapoint corresponds to a particular motif instance. (B) DeepLIFT vs. SHARPR correlation of the average motif match scores across all matches of one of the 1,934 different TF motifs. Each datapoint corresponds to a particular motif. (PDF) [file pone.0218073.s003.pdf]

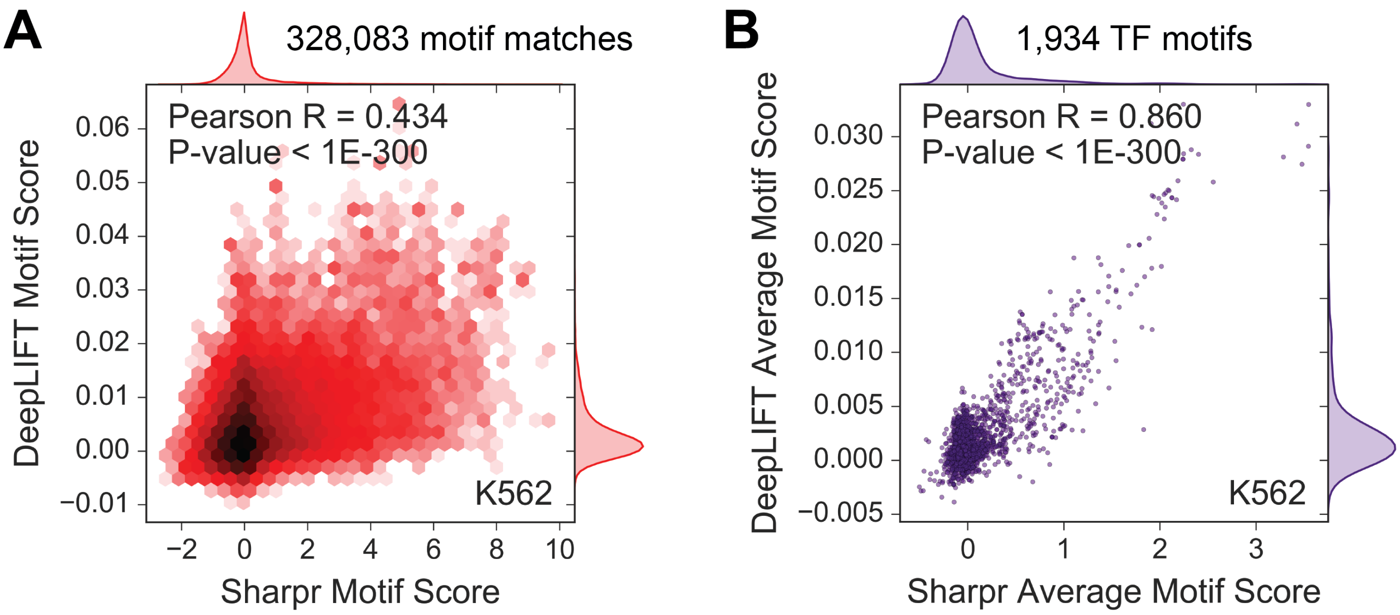

Supplementary Figure 3: Agreement between DeepLIFT and SHARPR nucleotide scores at transcription factor motif matches.
(A) Correlation between averaged DeepLIFT vs. averaged SHARPR nucleotide scores at each of the 328K motif matches that overlap at least one Sharpr-MPRA fragment. Each datapoint corresponds to a particular motif instance.
(B) DeepLIFT vs. SHARPR correlation of the average motif match scores across all matches of one of the 1,934 different TF motifs. Each datapoint corresponds to a particular motif.
