# Supplementary material for: Deciphering regulatory DNA sequences and noncoding genetic variants using neural network models of massively parallel reporter assays
Source: PLoS One. 2019 Jun 17;14(6):e0218073. doi: 10.1371/journal.pone.0218073 (PMC6576758; doi:10.1371/journal.pone.0218073)
Supplement: S4 Fig — (A) Boxplots showing distributions of LD-adjusted ISM scores (using the HepG2 minP model) for significant (P < 5×10^−8^) GWAS variants correlated with LDL cholesterol vs. insignificant (P > 0.1) GWAS variants. The significant variants are scored higher (P = 8.8 × 10^−9^). (B) Boxplots showing distributions of unadjusted ISM scores for significant vs. insignificant GWAS tag variants. Without accounting for linkage disequilibrium, there is no difference in scores between significant and insignificant variants. (C) Genome-wide correlation between LD-adjusted variant ISM scores and P-values of association of the variants with LDL cholesterol levels. (PDF) [file pone.0218073.s004.pdf]

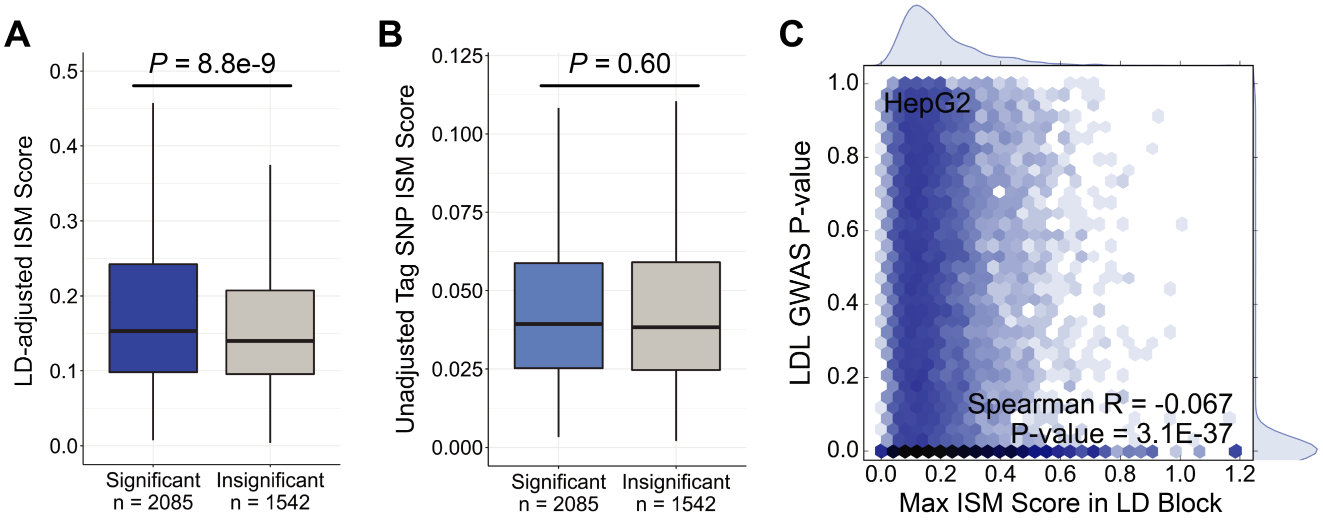

Supplementary Figure 4: LD-adjusted ISM scores offer some discriminating power between significant and insignificant GWAS variants.

(A) Boxplots showing distributions of LD-adjusted ISM scores (using the HepG2 minP model) for significant ($P < 5{\times}10^{-8}$) GWAS variants correlated with LDL cholesterol vs. insignificant ($P > 0.1$) GWAS variants. The significant variants are scored higher ($P = 8.8 \times 10^{-9}$).

(B) Boxplots showing distributions of unadjusted ISM scores for significant vs. insignificant GWAS tag variants. Without accounting for linkage disequilibrium, there is no difference in scores between significant and insignificant variants.

(C) Genome-wide correlation between LD-adjusted variant ISM scores and P-values of association of the variants with LDL cholesterol levels.
