# Supplementary material for: Deciphering regulatory DNA sequences and noncoding genetic variants using neural network models of massively parallel reporter assays
Source: PLoS One. 2019 Jun 17;14(6):e0218073. doi: 10.1371/journal.pone.0218073 (PMC6576758; doi:10.1371/journal.pone.0218073)
Supplement: S5 Fig — (A) HepG2 epigenetic landscape in the genomic neighborhood of rs174593. (B) Sorted FADS2 expression boxplots for a number of different tissue types from the GTEx consortium [60]. (C) FADS2 expression vs. rs174593 genotype for three tissues that express FADS2 from (B). Notably, aorta and esophagus cells display greater epigenomic activity at the rs174593 locus (data from Roadmap, not shown) and have stronger eQTL signal, suggesting that rs174593 may act as a disease variant through these (or other) tissues. (PDF) [file pone.0218073.s005.pdf]

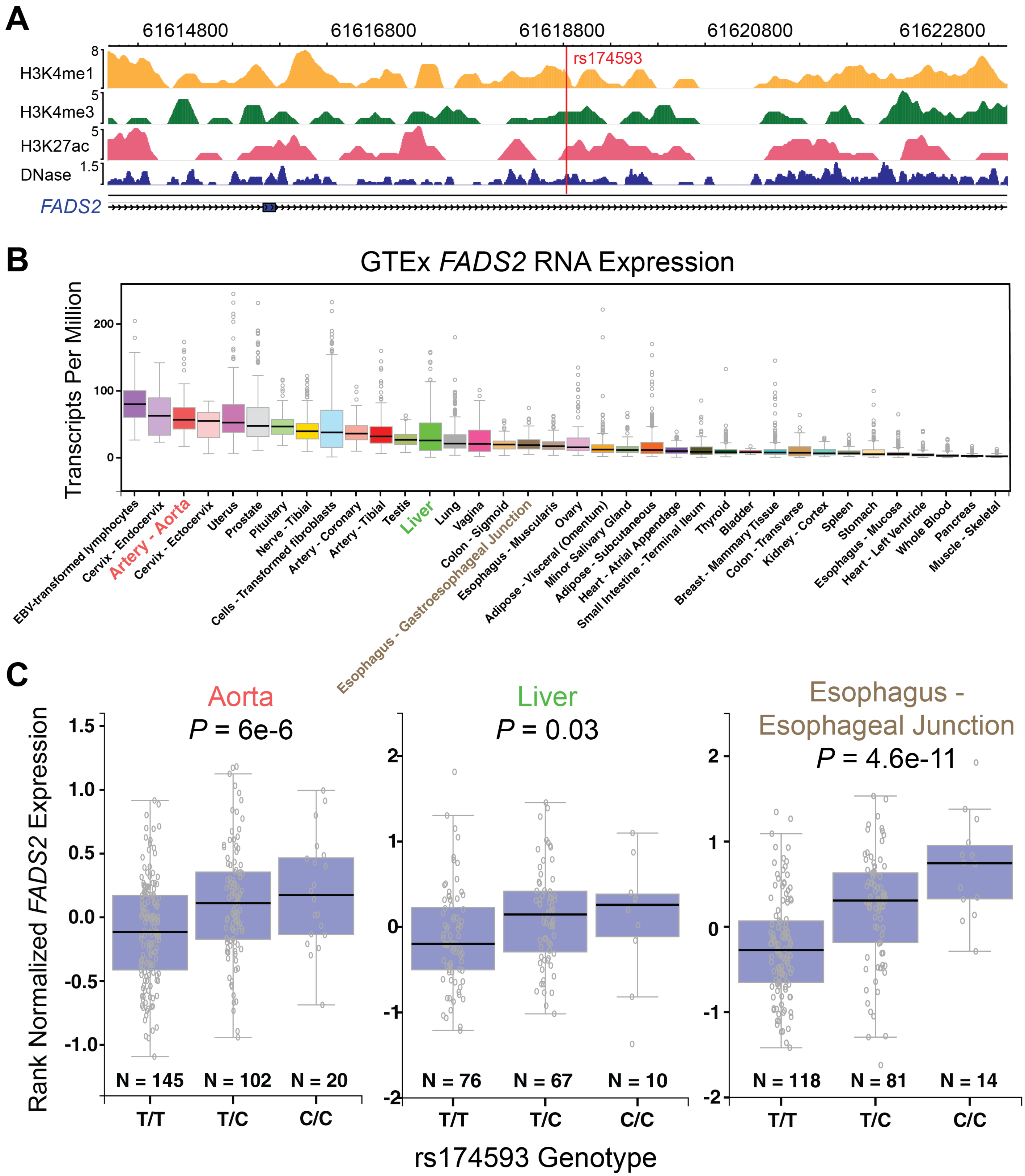

Supplementary Figure 5: Further evidence for rs174593 as a regulator of *FADS2* expression.
(A) HepG2 epigenetic landscape in the genomic neighborhood of rs174593.
(B) Sorted *FADS2* expression boxplots for a number of different tissue types from the GTEx consortium[58].
(C) *FADS2* expression vs. rs174593 genotype for three tissues that express *FADS2* from (B). Notably, aorta and esophagus cells display greater epigenomic activity at the rs174593 locus (data from Roadmap, not shown) and have stronger eQTL signal, suggesting that rs174593 may act as a disease variant through these (or other) tissues.
